# Neuromorphic Light‐Responsive Organic Matter for *in Materia* Reservoir Computing

**DOI:** 10.1002/adma.202501813

**Published:** 2025-05-13

**Authors:** Federico Ferrarese Lupi, Mateo Rosero‐Realpe, Antonio Ocarino, Francesca Frascella, Gianluca Milano, Angelo Angelini

**Affiliations:** ^1^ Advanced Materials Metrology and Life Science Division Istituto Nazionale di Ricerca Metrologica (INRiM) Strada delle Cacce 91 Torino 10135 Italy; ^2^ Department of Applied Science and Technology (DISAT) Politecnico di Torino C.so Duca degli Abruzzi 24 Torino 10129 Italy

**Keywords:** adaptive materials, *in materia* reservoir computing, intelligent materials, light‐responsive polymers, neuromorphic materials, physical reservoir computing

## Abstract

Materials able to sense and respond to external stimuli by adapting their internal state to process and store information, represent promising candidates for implementing neuromorphic functionalities and brain‐inspired computing paradigms. In this context, neuromorphic systems based on light‐responsive materials enable the use of light as information carrier, allowing to emulate basic functions of the human retina. In this work it is demonstrated that optically‐induced molecular dynamics in azopolymers can be exploited for neuromorphic‐type of data processing in the analog domain and for computing at the matter level (i.e., *in materia*). Besides showing that azopolymers can be exploited for data storage, it is demonstrated that the adaptiveness of these materials enables the implementation of synaptic functionalities including short‐term memory, long‐term memory, and visual memory. Results show that azopolymers allow event detection and motion perception, enabling physical implementation of information processing schemes requiring real‐time analysis of spatio‐temporal inputs. Furthermore, it is shown that light‐induced dynamics can be exploited for the *in materia* implementation of the unconventional computing paradigm denoted as reservoir computing. This work underscores the potential of azopolymers as promising materials for developing adaptive, intelligent photo‐responsive systems that mimic some of the complex processing abilities of biological systems.

## Graphical abstract

In this work we show that light‐responsive adaptive organic matter can store and process information at the matter level, and emulate neuromorphic functionalities such as short term memory, long term memory and visual memory. Besides demonstrating that material dynamics can be exploited for spatio‐temporal event detection and motion perception, we show that these adaptive photo‐responsive organic systems can be exploited for hardware implementation of physical reservoir computing.

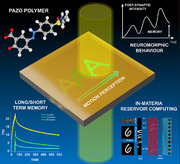

## 1 Introduction

Understanding and mimicking information processing capabilities of living systems has gained growing importance in science and technology.^[^ ^1^ ^]^ This interest stems from the remarkable efficiency of biological systems in handling complex tasks like classification, pattern recognition, and motion perception. In parallel with great achievements of machine learning algorithms based on neural networks at the software level, the interest in emulating the effectiveness of biological systems gained momentum since computing hardware technologies now require alternative approaches to overcome the inherent limitations of traditional schemes based on the Von Neumann architecture.^[^ ^2^ ^]^

Inspired by the adaptiveness of biological systems, neuromorphic computing aims to realize new computational platforms emulating the effectiveness of our brain through the development of new materials and devices.^[^ ^3^ ^]^ In this framework, the concept of “intelligent matter”, i.e., matter capable of interacting with the external environment by changing its internal structure in response to external stimuli, offers the potential to develop new class of physical substrates for brain‐inspired computing,^[^ ^4^ ^]^ where information processing and computing rely on the inherent physics of materials and devices.^[^ ^5^ ^]^ In this context, the dynamic response of materials and devices to external stimuli can be exploited for computing directly at the matter level – *in materia* – in the framework of the reservoir computing (RC) paradigm.^[^ ^6^, ^7^, ^8^, ^9^, ^10^, ^11^, ^12^, ^13^ ^]^

In recent years different approaches have been explored to incorporate neuromorphic functionalities into optical systems.^[^ ^14^ ^]^ These approaches include the integration of optical phase change materials (PCMs),^[^ ^15^, ^16^, ^17^ ^]^ optical resonators,^[^ ^18^ ^]^ optoelectronic devices based on Van der Waals (VdW) heterostructures^[^ ^19^, ^20^ ^]^ or hybrid organic‐inorganic systems exhibiting light‐dependent electrochemical doping that modulates the electrical response.^[^ ^21^, ^22^, ^23^, ^24^, ^25^, ^26^ ^]^ In this framework, light‐responsive materials are of particular interest to develop a novel class of optical devices for applications requiring simultaneous detection and processing of spatiotemporal visual stimuli, such as visual memory, pattern recognition, and motion perception.^[^ ^27^, ^28^ ^]^ Comprehensive reviews have been recently published to enumerate and compare these different approaches as well as discussing the underlying physical and chemical effects driving their functionalities.^[^ ^29^, ^30^, ^31^ ^]^ While these approaches hold great promises for neuromorphic applications, they still face significant challenges and limitations. PCMs are the most promising materials for integrated photonic circuits with neuromorphic processing capabilities,^[^ ^32^ ^]^ as they show excellent stability of non‐volatile states with multilevel storage capabilities^[^ ^33^ ^]^ and are CMOS compatible.^[^ ^34^ ^]^ However, the non‐volatile nature of the phase change hinders the use of PCM in applications where volatile memory is required, as in the case of temporal information processing. Additionally, PCMs pose significant fabrication and scalability challenges due to the high energy required for transitions.^[^ ^35^ ^]^ VdW heterostructures have been successfully employed as sensing devices incorporating neuromorphic functionalities (the so‐called in‐sensor computing).^[^ ^36^, ^37^ ^]^ VdW heterostructure show both volatile and non‐volatile processes that make them suitable candidates for mimicking short‐term (timescale 10^−1^–10 s) and long‐term plasticity effects (> 10^2^ s).^[^ ^38^ ^]^ Despite great advancements in the fabrication of high‐quality 2D materials, VdW heterostructures still pose significant fabrication and scalability challenges, and exhibit inherent limitations related to material stability, reproducibility, and high sensitivity to defects and environmental conditions.^[^ ^38^ ^]^ The class of hybrid organic–inorganic neuromorphic devices includes a large variety of different materials and devices, from halide perovskites^[^ ^39^ ^]^ to organic field effect transistors^[^ ^40^, ^41^ ^]^ Hybrid organic–inorganic systems such as the one reported by Chen and coworkers^[^ ^25^ ^]^ have been characterized for their synaptic response but so far there isn't an experimental proof of computational capabilities. Additionally, the multiple stacked layers of materials needed to achieve neuromorphic functions leads to complexity in the fabrication process, that results in large device‐to‐device variation that still hinders the use of this class of devices in practical applications.^[^ ^42^ ^]^ Moreover, when matrices of top‐down fabricated optoelectronic devices are used to process spatially distributed information, as for image recognition or motion perception, the required pixelization reduces the photosensitive surface and consequently the spatial resolution and density of data that can be stored and processed.^[^ ^15^, ^17^, ^26^, ^28^, ^43^, ^44^ ^]^

Among organic optoelectronic devices, azobenzene‐based organic field effect transistors are a promising class of photo‐responsive neuromorphic devices, due to the strong modulation of the electronic properties of azobenzene molecules when irradiated by proper light.^[^ ^45^, ^46^, ^47^ ^]^ However, these types of devices often require specific synthesis processes^[^ ^46^, ^47^ ^]^ or precise grafting and stacking of different materials,^[^ ^45^ ^]^ and don't exploit the full light‐driven molecular dynamics.

In this work, we report on neuromorphic‐type of data processing and *in materia* computing by exploiting the light‐induced molecular dynamics in a continuous azopolymer thin film. By exploiting the optically induced birefringence, i.e., the optical anisotropy arising from the molecular alignment fostered by polarized light,^[^ ^48^ ^]^ we show that the optical dynamic response of these materials can be exploited for emulating a wide range of neuromorphic functionalities, including short‐term memory, long‐term memory, and visual memory effects. In this context, we also illustrate that the adaptiveness of azopolymers can be exploited for spatio‐temporal event detection and visual motion perception tasks. Furthermore, we demonstrate the possibility of exploiting information processing capabilities of the azopolymer for *in materia* implementation of RC, as demonstrated by solving the handwritten digit Modified National Institute of Standards and Technology (MNIST) benchmark task.

## 2 Light‐Driven Molecular Dynamics

Azobenzene‐containing molecular compounds are well‐known for their high responsivity to light, which triggers molecular transition that results in light‐induced birefringence^[^ ^49^, ^50^ ^]^ or light‐driven mass migration.^[^ ^51^, ^52^, ^53^, ^54^ ^]^ While these mechanisms have been extensively studied for optical memory applications, the spontaneous molecular rearrangement caused by thermal fluctuation has hindered the path toward reliable optical memory systems.^[^ ^55^, ^56^ ^]^

**Figure 1:**
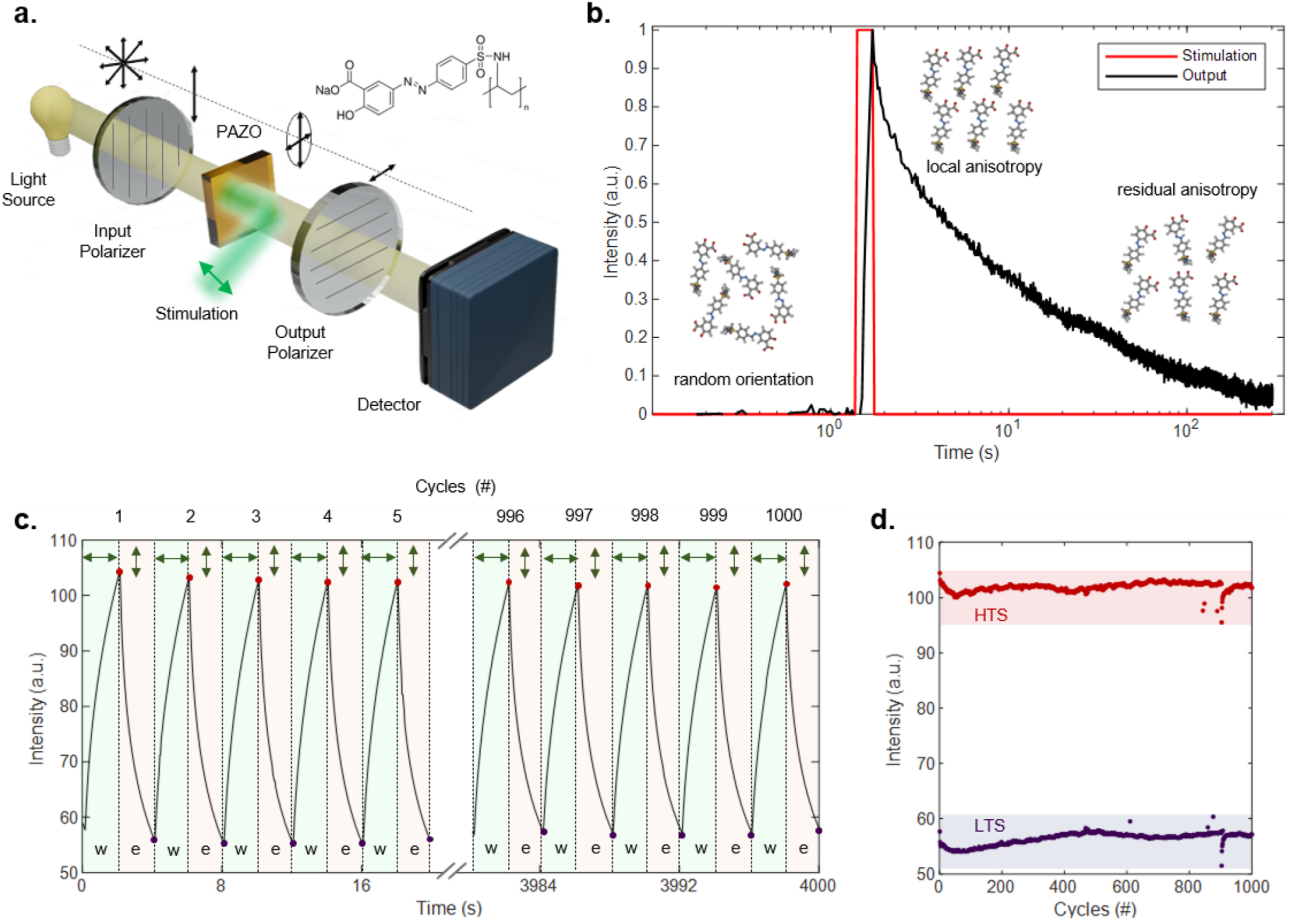
Light‐responsive organic matter. a) Scheme of the readout setup: a PAZO (chemical structure in the inset) thin film is included in a cross‐polarized white light imaging system. A polarized laser beam (λ = 532 nm) locally stimulates the PAZO and induces birefringence. b) Temporal profile of the intensity of the laser beam (red) that stimulates the birefringence in PAZO and time evolution of the light intensity at the detector (output – black). The *x*‐axis is logarithmic. A sketch of the molecular arrangement helps to associate the output intensity to the induced anisotropy. c) Output light intensity under 1000 write‐erase cycles (period 4 s, duty cycle 50%) of stimulation with alternating orthogonal polarization states. d) Endurance of the system showing the stability of the HTS and LTS under 1000 writing and erasing cycles. All the recorded values are within the red band (HTS) and the purple band (LTS).

Light‐induced birefringence in poly[1‐[4‐(3‐carboxy‐4‐hydroxyphenyl‐azo) benzene sulfonamido]‐1,2‐ethanediyl, sodium salt] (PAZO), a commercially available azopolymer, is here exploited for both processing and storing information at the matter level. In this context, a linearly polarized laser beam stimulates the material, while a cross‐polarized white light imaging system allows to read the internal state of the system (**Figure** **1**a; details in Experimental Section and Figure S1, Supporting Information). The information about the molecular configuration is indeed encoded in the polarization state of light passing through the material, and the linear polarization filter before the detector allows to convert this information into light intensity on the CMOS camera. The linearly polarized laser light with a wavelength falling within the absorption band of PAZO (Figure S2, Supporting Information) stimulates the light‐responsive material by inducing birefringence. While high power densities can induce mass‐migration effects (Figure S3, Supporting Information), the power densities exploited in our work have been observed to result in negligible material degradation. The stimulated region alters the polarization state of white light passing through it, thus modulating the light intensity transmitted through the output polarizer and read by the detector. An example of PAZO dynamics for a 1 µm thick film under pulse light stimulation at λ = 532 nm is reported in Figure 1b (details on the thin film thickness in Figure S4, Supporting Information). An analysis of the influence of the film thickness on the material optical response revealed that a thicker film results in a stronger optical response in terms of variation of the light transmitted intensity (details in Figure S5, Supporting Information), in accordance with previous works.^[^ ^48^ ^]^

Before stimulation (material in the pristine transmittance state), PAZO molecules are randomly oriented, thus the polarization state of light passing through it is not affected, and the cross‐polarizers minimize the transmitted light intensity. When stimulated by a laser pulse (red line, stimulation power density P = 8.3 nW µm^−2^, pulse duration t_ON_ = 500 ms) PAZO molecules tend to align orthogonally to the laser polarization and the molecular alignment induces optical anisotropy that results in progressively increasing transmitted white light intensity. Therefore, the internal state of the system gradually goes from the initial low transmittance state (LTS) toward a high transmittance state (HTS). After the end of laser stimulation, molecules spontaneously tend to relax to the initial (pristine) isotropic orientation, leading to a spontaneous gradual relaxation of the system from the HTS toward the initial LTS. A description of the molecular kinetics of azopolymers under polarized light illumination and an empirical explanation for the two exponential terms has been discussed in literature.^[^ ^57^, ^58^ ^]^ Briefly, two mechanisms contribute to the overall birefringence: *i)* a fast process associated with the cis‐to‐trans (potentiation) and trans‐to‐cis (relaxation) transitions that results in preferential orientation of the PAZO molecules when triggered by linearly polarized light, and *ii)* a slower thermally activated re‐orientational motion of molecules that randomizes the average molecular orientation. These two mechanisms result in double exponential potentiation and relaxation/erasing dynamics, as detailed in Supplementary Note N1. Notably, birefringence in PAZO can be induced using a broad range of excitation wavelengths,^[^ ^59^ ^]^ where shorter wavelengths result in longer decay constants after stimulation (details in Figure S6, Supporting Information). Although our study is focused on PAZO, it is worth noticing that similar optical responses can be obtained in other azobenzene‐based polymeric systems (an experimental example of the dynamic response of the poly Disperse Red 1 Methacrylate (pDR1M system is reported in Figure S7, Supporting Information), and that the dynamics can vary depending on the polymeric blends properties.^[^ ^57^ ^]^ Note that if the stimulation is more intense or prolonged in time, the system can retain information about the stimulation for a long time (> 6 h) and relax to a state with residual anisotropy (Figure S7, Supporting Information).

Besides spontaneous relaxation of the transmittance state over time, the adaptiveness of the PAZO response allows to actively erase the previously stored information by changing the polarization state of the input light stimulation from linear to circular or orthogonal polarization (Figure S8a–c, Supporting Information). Circular polarization randomizes the molecular orientation bringing the system back to its pristine state, while rotating the polarization of the stimulation rotates the molecules orientation thus modifying the orientation of fast and slow axes with respect to the initial white light polarization state. High reproducibility of the system response over repeated write/erase cycles has been observed (Figure 1c), as testified by the endurance test reporting LTS and HTS found to be stable for > 1000 cycles (Figure 1d).

## 3 Light‐Induced Synaptic Plasticity and Visual Memory

**Figure 2:**
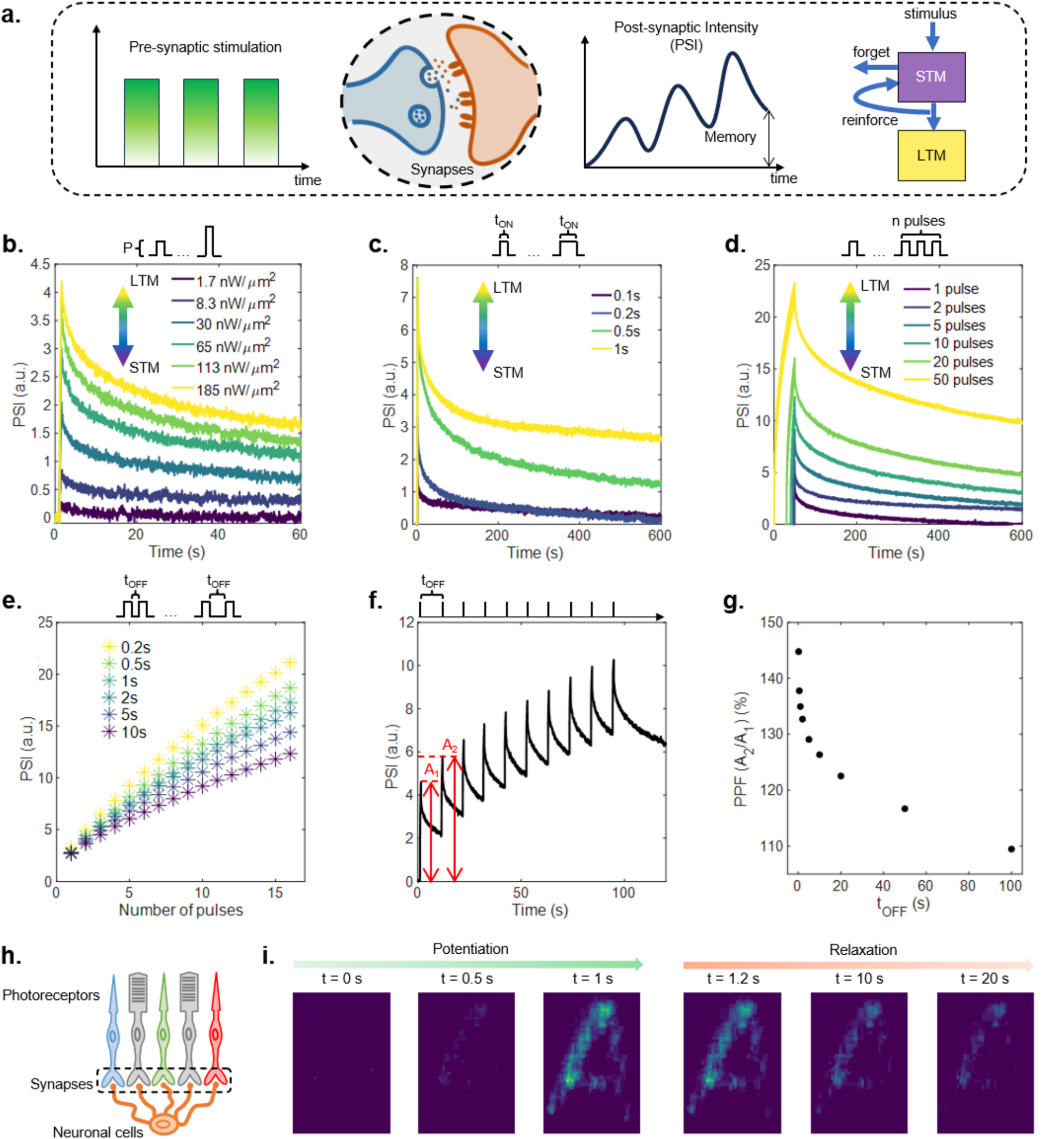
Synaptic functionalities. a) Sketch of a synaptic connection stimulated by optical pulses (pre‐synaptic stimulation) that enhances the PSI. On the right, a scheme of the transition between STM and LTM. b–d) Time evolution of PSI showing STM to LTM transition by: (b) increasing P (t_ON_ = 200 ms), (c) increasing t_ON_ (P = 8.3 $\frac{nW}{\mu m^{2}}$), (d) increasing number of pulses (P = 8.3 $\frac{nW}{\mu m^{2}}$, t_ON_ = 500 ms, t_OFF_ = 500 ms). e) Effect of the time delay t_OFF_ between 16 pre‐synaptic pulses on the peak intensities of PSI (P = 8.3 $\frac{nW}{\mu m^{2}}$, t_ON_ = 500 ms). f) Time evolution of PSI when stimulated by 10 optical pulses (P = 8.3 $\frac{nW}{\mu m^{2}}$, t_ON_ = 500 ms, t_OFF_ = 10 s). The PSI peak values after the first and second pulses are labeled as A_1_ and A_2_. g) PPF measured for different t_OFF_. h) Sketch of the outer layer of human retinal structure. i) Time sequence of potentiation and relaxation of a distributed intensity pattern stimulating the PAZO. Images are selected frames extracted from Video S1 (Supporting Information).

The dynamic response of PAZO can be exploited to emulate the short‐term plasticity of biological synapses, characterized by progressive enhancement of the signal transmission during stimulation (potentiation) followed by spontaneous relaxation to the initial ground state after the end of stimulation.^[^ ^60^ ^]^ A time sequence of stimulation pulses (pre‐synaptic stimulation) will result in a potentiation of the Post‐Synaptic Intensity (PSI) and eventually lead to long‐lasting visual memory, if the stimulation is sufficiently reinforced or otherwise to forget the information as a consequence of the spontaneous relaxation (**Figure** **2**a). In these terms, the system enables us to emulate short‐term memory (STM) and long‐term memory (LTM) effects depending on the input light stimulation in terms of laser pulse density power, pulse duration, pulse number, and temporal distance between pulses, as reported in Figure 2b–g.

An increase of P of a single stimulation pulse results in an increase of PSI, as reported in Figure 2b (examples of stimulation at different wavelengths and with different power densities is reported in Figure S9, Supporting Information). While the PSI tends to relax back to the pristine value in case of low P (STM), the tendency to decay to a residual value increases when stimulation power density P is increased (LTM). A similar transition from STM to LTM is observed by increasing the pulse duration (t_ON_) as reported in Figure 2c shows the evolution of PSI. Indeed, while short pulses (t_ON_ = 100 and 200 ms) produce an increase in the PSI that returns to the initial state in few minutes, longer pulses (t_ON_ = 500 ms, 1s) results in a residual value of PSI higher than the initial value is observed.

Figure 2d shows the PSI evolution of the PAZO transmittance under stimulation with pulse trains with increasing number of pulses. While a single pulse produces a STM effect and the system relaxes back to the pristine state in ≈10 min a transition between STM to LTM is observed by increasing the number of pulses. Similarly to biological synapses, the temporal correlation of input signal (*i.e*., the temporal delay between stimulation pulses) influences the final state of the system: Figure 2e shows the peak value of PSI after each stimulation pulse when the time delay (t_OFF_) between the pulses varies from 200 ms to 10 s, showing higher potentiation for higher correlated stimulation signals. The gradual increase of transmittance over pulses, that results from the competition between potentiation during laser irradiation and spontaneous relaxation in between light pulses (Figure 2f) can be exploited to emulate typical features of short‐term plasticity features such as Pair Pulse Facilitation (PPF).^[^ ^61^ ^]^ Figure 2g reports PPF evaluated as the ratio between the PSI peak after the second pulse (A_2_) and the peak after the first pulse (A_1_), revealing an increase of PPF by increasing the temporal correlation between pair pulses.

The synaptic‐like response of PAZO to light stimulation can be exploited also for the emulation of visual perception and visual memory features typical of biological systems. Vision in living organisms is a complex phenomenon that involves photosensitive cells acting as photoreceptors connected to the neural system via synapses (sketch of the outer layer of mammalian retina in Figure 2h).^[^ ^62^ ^]^ A matrix of photosensitive cells (rods and cones) enables the perception of light intensity distributions (i.e., images). The synaptic connection to the first neuronal layers enables the pre‐processing and memorization of images, and different type of visual memories can occur, from the very short‐term iconic memory (few hundreds of ms) to Visual STM and Visual LTM.^[^ ^63^ ^]^ Due to the local nature of light‐induced birefringence, the dynamic response described above for a point‐like stimulation pulse can be extended to spatially distributed intensity light patterns to mimic the perception and persistence of images on the retina, that are the basic functions for visual memory (details in Figure S10, Supporting Information). In this framework, PAZO enables the storage of spatial patterns both in the short and long terms depending on the input stimulation. Figure 2i (and related Video S1, Supporting Information) shows an example of an “A” pattern stored in the short term, while an example of the same pattern stored in the longterm is reported in Figure S11 (Supporting Informationand related Video S2, Supporting Information), where it is possible to observe that the long‐term stored pattern can be eventually actively erased partially or completely through appropriate stimulation.

## 4 Event Detection and Visual Motion Perception

**Figure 3:**
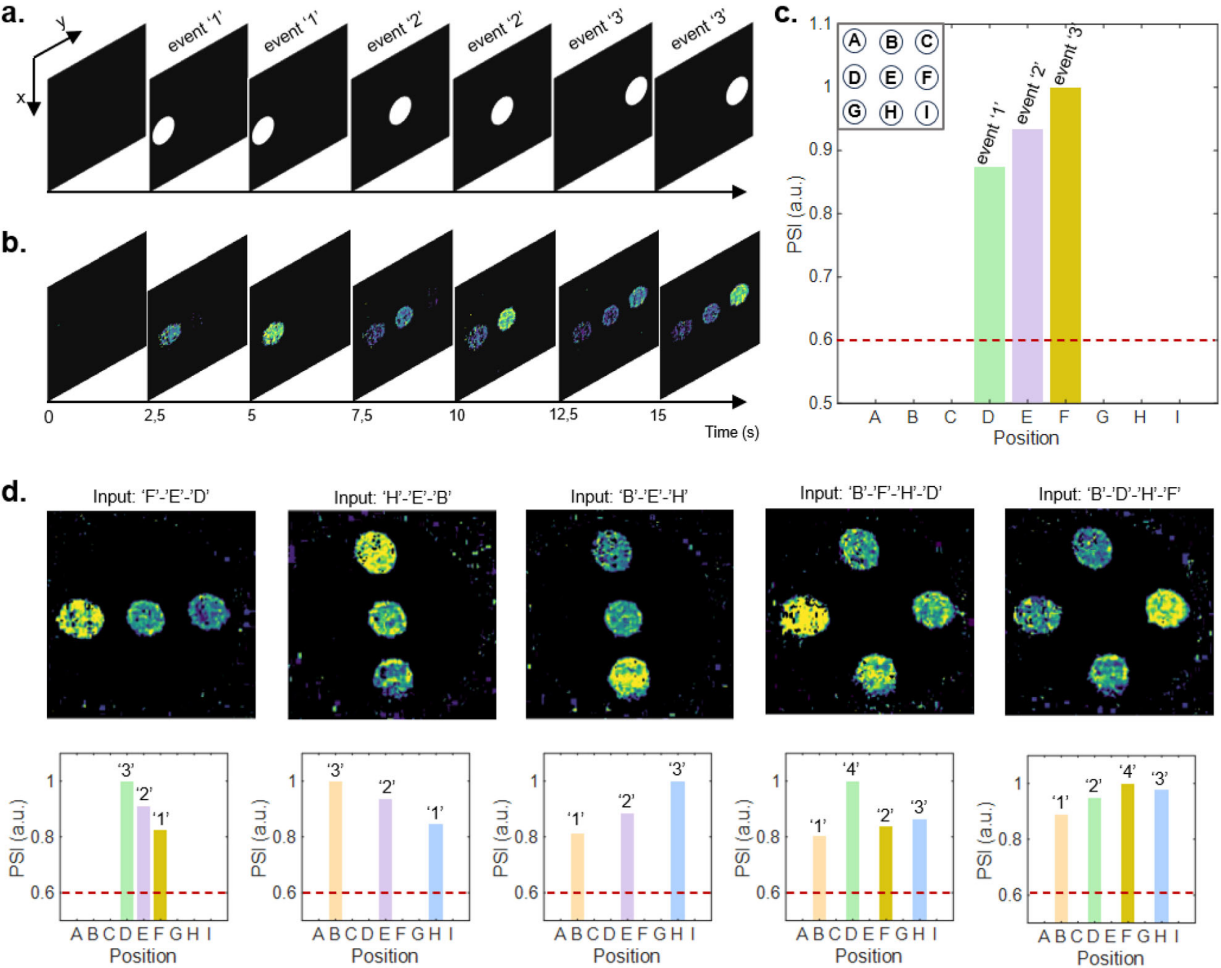
Event sequence detection a) Schematic representation of a sequence of events (white spot) happening in different locations and at different times. b) Corresponding PSI was detected at different times. c) PSI output intensity measured at the last frame (t = 15 s). In the inset above, the space is discretized in 9 positions labeled with letters (A–I). The PSI at each position is reported in the bar plot. The red dashed line indicates the event threshold value. d) Different event sequences corresponding to distinct sequences of events, are labeled accordingly. For each sequence, the PSI value at each position is reported in the bar plot underneath.

Spatiotemporal processing capabilities of the system allow detecting the history of events that occurred in different spatial regions at different times by analyzing the final state of the system. **Figure** **3**a schematizes an example of temporal sequence of events (white spot) occurring in different spatial positions. Here, the temporal sequence of 3 events (represented as a light pulse with P = 0.15 µW µm^−2^, t_ON_ = 5s) labeled ‘1′, ‘2′, and ‘3′ separated by 5 s each and occurring in three different spatial coordinates is reported. The corresponding spatiotemporal evolution of the system is reported in Figure 3b (complete video in Video S3, Supporting Information). Consequently, between the potentiation of stimulated areas and their fading dynamics, the final state of the system allows both to identify the event location and their temporal sequence (Figure 3c). Here, bar plots indicate the intensity of each specific location in the final state (red dashed line indicates the event threshold intensity), where the lowest PSI corresponds to the furthest event in time thus enabling temporal ordering of events (details in Figure S13, Supporting Information). Examples of the system response to different sequences of events are reported in Figure 3d, where the final state of the system is reported along with corresponding transmission intensity bar plots. As can be observed, in all cases the analysis of the final internal state of the system allows to correctly reconstruct the spatiotemporal history of events.

**Figure 4:**
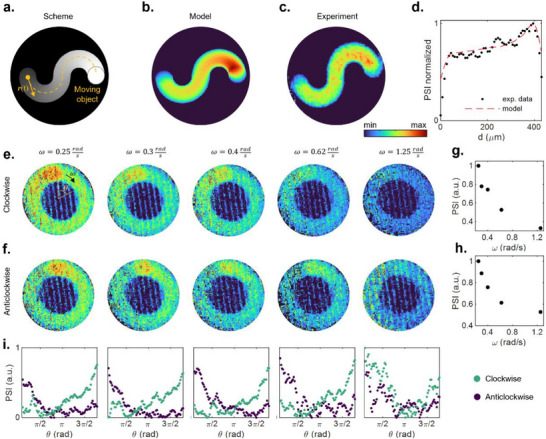
Visual Motion Perception a) The fading white spot corresponds to a continuously moving object in the image plane. ***r*** *(t)* represents the position vector that changes over time, and the dashed yellow line is the overall trajectory. b) Simulated and c) measured PSI trace of the moving object extracted at the end of the trajectory (full Video in Video S4, Supporting Information). d) Simulated (red dashed line) and experimental (black dotted line) PSI extracted from (b) and (c) respectively along the trajectory in (a), where *d* is the distance from the initial point along the object trajectory. b) Temporal sequence of frames corresponding to the PSI when the stimulation follows a clockwise circular path with *ω* = 0.25 rad s^−1^. e,f) From left to right, false color PSI images of clockwise (e) and anticlockwise (f) continuously moving stimulation at different angular speeds. g,h) Integrated PSI along the path as a function of *ω* for clockwise (g) and anticlockwise (h) directions. i) PSI was extracted along the stimulation path for each angular speed for the clockwise (green dots) and anticlockwise (purple dots) directions.

So far, we have considered temporally and spatially independent events, but the physical substrate allows perceiving a moving object in a spatially continuous domain. Motion perception is indeed the ability to detect and elaborate continuous variations in space and time of a light stimulus. As an example, we tested the ability of the material to perceive the information relative to a moving object (represented by a light spot) moving along an arbitrary trajectory (scheme in **Figure** **4**a). Figure 4b,c report the PSI trace left by an object moving along that trajectory (see Video S4, Supporting Information) details about the model in Experimental Section). Modeled and experimental PSI signals as function of the distance *d* traveled along the trajectory are reported in Figure 4d, showing good agreement between the model and the experiment. As can be observed, the moving object results in a continuous and spatially distributed memory trace that tends to progressively vanish over time due to the short‐term effects of the system. Since the local transmittance of the physical substrate depends on when the object passed in this spatial location, the analysis of a single frame can be exploited to retrieve information about the movement trajectory, direction, and speed of the object. As an example, Figure 4e,f show the PSI trace of clockwise (c) and anticlockwise (d) movements where the angular speed *ω* is progressively increased from left to right. The integrated PSI over the whole trace enables it to sort the moving objects by their angular speed (the higher the speed, the lower the residual PSI), as reported in Figure 4g,h (clockwise and anticlockwise movements respectively). The plots in Figure 4i on the other hand report the PSI versus the angular position *θ* for clockwise (green dots) and anticlockwise (purple dots), and the gradient of intensity allows to retrieve the direction of motion at the different speeds. Note that, for the given stimulation power density (P = 0.15 µW µm^−2^) and detection system, the signal‐to‐noise ratio (S/N) is strongly reduced at speeds higher than 1.25 rad s^−1^ and the distinction between the two directions becomes harder to retrieve. Faster moving objects could be detected by increasing the S/N (Figure S15, Supporting Information), although it is worth noticing that the relaxation dynamic limits the possibility to retrieve the movement of objects moving with arbitrary speeds, as increasingly faster objects require increasingly sensitive detection systems (details in Figure S16, Supporting Information).

## 5 *In Materia* Reservoir Computing

**Figure 5:**
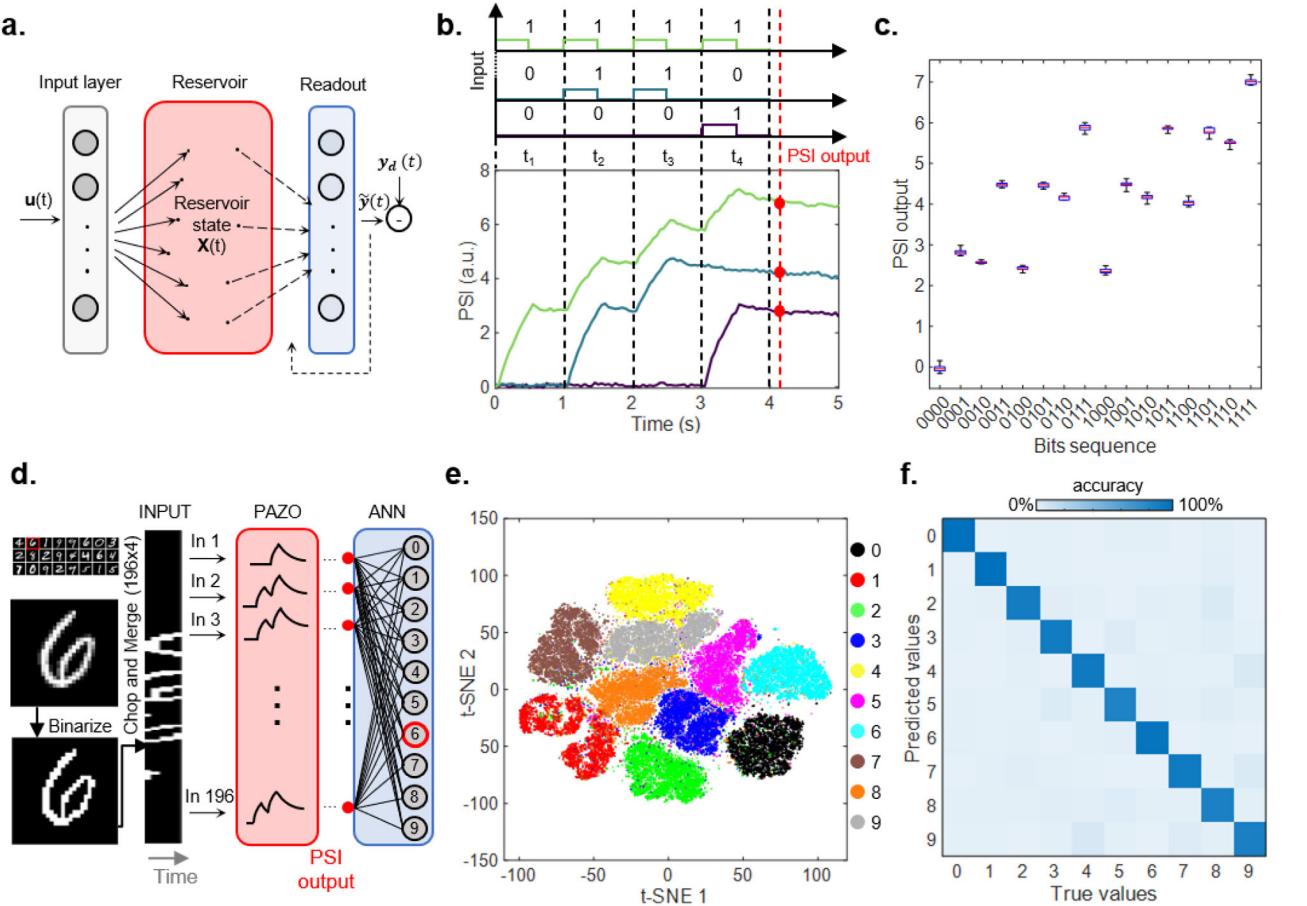
*In materia* reservoir computing. a) Sketch of the RC framework where the input is transformed by the reservoir into a feature space that is then analyzed by a readout that is the only part to be trained. b) Time sequences of stimulation pulses that encode 4‐bit sequences and the corresponding PSI outputs. The red dots indicate the PSI values read 100 ms after the last bit (red dashed line). c) Boxplot of the PSI output corresponding to 10 repetitions of each of the 16 different bit sequences. The red line is the median value. The upper and lower edges of the box are the upper and lower quartile, while the whiskers extend to the minima and maxima. d) Workflow for the implementation of the MNIST handwritten digits recognition task. The original 28 × 28 pixels images are binarized, chopped, and merged into aa 196 × 4 matrix corresponding to 196 sequences of stimulation pulses. The red box corresponds to the PAZO reservoir that nonlinearly transform the input stimulation into the PSI outputs that are then fed as input to the ANN. e) Visualization of the reservoir outputs embedding via t‐SNE algorithm. f) Confusion matrix of the predicted values when classifying the MNIST test set of 10000 handwritten digits, after training the read‐out with the MNIST training set of 60000 handwritten digits. The color bar represents the accuracy of prediction for each digit.

Nonlinear dynamics and short‐term memory capabilities of PAZO can be exploited for the physical (*in materia*) implementation of the RC unconventional computing paradigm.^[^ ^6^, ^7^ ^]^ RC is a computational framework where computation is divided in two parts, as schematized in **Figure** **5**a. The *reservoir* maps an input signal *u*(*t*) into a feature space represented by the reservoir state *x*(*t*) that is then analyzed by a *readout* that is the only part that needs to be trained by comparing the actual output. $\tilde{y}\left(t\right)$ of the system to the desired one *y_d_* (*t*). Here, we show that PAZO dynamics can be exploited to realize a physical reservoir that allows to nonlinearly map input signals (in terms of light stimuli) into a feature space that can be analyzed by a readout.

Figure 5b reports the response of the PAZO over time to different 4‐bit sequences mapped as trains of light input pulses. In particular. The 4‐bit sequences are encoded in a sequence of 4 timeframes (length of 1 s), where in each timeframe a pulse of 500 ms is applied or not in case of bit ‘1′ and ‘0′, respectively (details in Experimental Section). Here, the competition between potentiation and spontaneous relaxation results in a final state of the system (PSI output, read after 100 ms from the last timeframe) that depends on the specific bit sequence. The response of the system to all 4‐bit sequences is reported in Figure 5c, where box plots are constructed based on the system response to 10 repetitions of each input sequence (complete cycle‐to‐cycle time traces of sequences in Figure S17, Supporting Information). Notably, the output of the system is strongly different for each sequence, and the variability of the system response is substantially lower than the difference in between the system response to different sequences. To assess the device‐to‐device variability, the PSI output of each sequence was evaluated in four different points on the sample surface, revealing a device‐to‐device variability of ≈ 2.5% (Figure S18, Supporting Information), while cycle‐to‐cycle variability is within the measurement noise (Figure S19, Supporting Information and details in Experimental Section).

As a proof‐of‐concept of *in* *materia* reservoir computing, the acquired data are used to test the capability of the system on a benchmark pattern recognition task, namely the MNIST handwritten digit classification task, by exploiting the PAZO as physical reservoir (Figure 5d). For this purpose, the 28 × 28 digits from the MNIST dataset are binarized, chopped, and merged to form a 196 × 4 pattern (details in Experimental Section). This pattern with black or white pixels is then converted into a spatio‐temporal pattern composed of 196 pulse streams with four timeframes. The 4‐bit rows of the pattern represent a subset of 4‐bit sequences previously applied to the PAZO, where white pixel is represented by bit ‘1′ while black pixel is represented by bit ‘0′, and the system PSI output for each row is randomly extracted from the set of 10 measured transmittance values for a certain sequence (this allows to take into account of the cycle‐to‐cycle variability). In these terms, the final reservoir state to be passed to the readout for classification is represented by the ensemble of the 196 system PSI outputs, one for each 4‐bit sequence composing the binarized, merged, and chopped digit. These output signals are then exploited as input of a 196 × 10 fully connected artificial neural network (ANN) serving as the readout for classification (details in Experimental Section). The capability of the dynamic reservoir to separate different input signals is visualized through the t‐distributed stochastic neighbor embedding (t‐SNE) algorithm.^[^ ^64^ ^]^ Figure 5e shows the t‐SNE embedding of PAZO reservoir outputs, where data shows higher clusterization with respect to the same input data not processed by reservoir dynamics (details in Figure S20, Supporting Information). After readout training (details in Experimental Section, Figure S21, Supporting Information), the system can correctly classify input digits with an accuracy of ≈91.4% (confusion matrix in Figure 5d, details in Experimental Section and Figure S22, Supporting Information). A comparison with performances of other physical reservoirs on the MNIST benchmark task is reported in Table S1 (Supporting Information). Notably, training of the readout by considering different random choices of PSI outputs reveals that the variability of the PAZO response is not strongly affecting the accuracy, demonstrating robustness of the system (Experimental Section, details in Figure S23, Supporting Information).

## 6 Discussion and Conclusions

Our results show that a commercial azopolymer thin film can be exploited to realize a cost‐effective platform for neuromorphic computing. The system requires only a single fabrication step, consisting of spin‐coating a glass substrate with an azopolymer‐containing solution, eliminating the need for cleanroom facilities or complex lithographic processes. The low‐cost fabrication and the deposition flexibility of PAZO polymers allow the realization of hybrid devices based on PAZO polymers coupled with conventional electronic and photonic architectures to enhance the computational efficiency. Their compatibility with organic photodetectors, waveguides, and plasmonic nanostructures allows for optical signal preprocessing, while their use with flexible and transparent substrates supports hybrid systems for real‐time, *in materia* computing in embedded or edge AI platforms.

Unlike top‐down fabricated discrete optoelectronic devices,^[^ ^22^, ^25^, ^26^, ^43^, ^44^ ^]^ this system endows the processing of the input signal not only in the continuous temporal domain but also in the continuous spatial domain. This represents an important advantage not only for processing input signals in the form of images with higher spatial resolution, but also for high‐resolution event detection and visual motion detection.

A key feature of this system is that the material's internal state is encoded in the polarization state of light transmitted through it. In our detection setup this vectorial state is accessed by projecting the polarization vector onto a scalar quantity, i.e., the intensity of light that represents the physical observable. However, the full vectorial information may be retrieved by adopting other detection schemes to retrieve the full Jones matrix.^[^ ^65^ ^]^

Importantly, the azopolymer dynamics enables the implementation of both STM and LTM. While STM represents a key aspect for temporal processing of the input signal, LTM enables long‐term data storage in the same physical substrate with the possibility to actively reset the internal state of the material. Notably, the dynamic response of the system can be tuned depending on the input stimulation in terms of power density, duration, number, and temporal correlation of input stimuli. This adaptability ensures the matching between device dynamics and input signal to be processed. In this context, we show that nonlinear dynamics and STM capabilities can be exploited for physical reservoir computing, a computing paradigm with reduced training costs. Here, the robustness of the computing system is ensured by the high reproducibility of azopolymer dynamics. It is worth mentioning that the analogue PAZO dynamics can be leveraged to explore also different implementation strategies of physical reservoir computing. For instance, system dynamics can allow the implementation of purely time‐dependent computing tasks, such as time‐series prediction, by exploiting a time multiplexing scheme.^[^ ^66^ ^]^ In this approach, an irradiated area of azopolymers serves as a single dynamical node with delayed feedback for temporal processing of the input signal.

In conclusion, we believe that our findings unravel the potential of azopolymers to add information processing ability at the matter level in optical devices toward the realization of neuromorphic optical systems. The proposed approach paves the way toward a new paradigm in visual computing and image sensing, moving the spatio‐temporal information processing at the matter level and thus reducing the computational effort to process data and extract features.^[^ ^67^ ^]^ Considering the wideness of the class of azopolymers,^[^ ^68^ ^]^ additional functionalities such as biocompatibility,^[^ ^69^, ^70^ ^]^ wavelength selectivity^[^ ^71^ ^]^ and environmental sensitivity^[^ ^72^ ^]^ might be explored, and innovative azopolymers can be synthesized ad hoc to tailor the molecular dynamics and add functionalities.^[^ ^73^, ^74^ ^]^

## 7 Experimental Section

### Sample Preparation

A solution of commercially available PAZO (Merck) was prepared as follows. PAZO powder was dissolved in methanol at a concentration of 100 mg ml^−1^. To promote dissolution, mechanical stirring, and sonication for a few minutes was performed. When the solution results homogeneous, an amount of 150 µl of PAZO methanol was casted onto a glass substrate, previously washed in acetone, rinsed with isopropanol, and dried with a nitrogen flow. Just after depositing the polymer solution in the substrate, the sample was spin‐coated at 800 rpm for 1 min to obtain a uniform 1 µm thick film (Figure S3, Supporting Information) above the substrate. The sample was then dried in an oven at 60 °C for 2 h, until the solvent evaporates (3 h at 60 °C).

### Experimental Setup

A detailed scheme of the experimental setup was reported in the Supporting Information (Figure S1, Supporting Information). The cross‐polarized imaging microscope was equipped with a broadband halogen lamp (HL – High‐Intensity Fiber‐Coupled Illuminator from Thorlabs) that was collimated and then filtered by a linear polarizer (LP1 – Linear Polarizer LPVIS100 from Thorlabs) before illuminating the PAZO sample. On the other side of the sample, a 10x objective (Olympus PlanC 10x NA 0.25) or 5x objective (Olympus PlanC 5x NA 0.12) (labeled as OBJ in Figure S1, Supporting Information) collects the transmitted white light that passes through a 50:50 beam splitter (BS – non polarizing beam splitter for Vis light from Thorlabs) and was filtered by a linear polarizer (LP2) orthogonal to LP1 and by a long pass filter (LPF550 – Longpass Filter, Cut‐On Wavelength: 550 nm from Thorlabs). A biconvex lens produces the image of the sample surface on a CMOS camera (CMOS – IDS UI‐3260CP‐M‐GL Rev.2). In the experiments, the “zero” level of light intensity was obtained by subtracting the residual intensity transmitted through the imaging system and recorded by the camera when the material is in its pristine state. The stimulation was provided by a laser source at 532 nm (LS –Gem 532 from Laser Quantum Ltd.) The beam emitted was gaussian and linearly polarized, and it can pass through a programmable polarization rotator (PR – LCR1‐532 from Thorlabs) or a programmable wave plate retarder (WP – LCC1111T‐A from Thorlabs) to rotate the polarization or convert it into circular polarization. Both of them could controlled with the same liquid crystal controller (LCC25 from Thorlabs). The beam was then expanded by a beam expander (BE1) composed by two biconvex lenses (L1 – focal length 50 mm and L2 – focal length 150 mm) before illuminating the display of a Digital Micromirror Device (DMD – DLP6500 from Texas Instrument) that allows to shape the wavefront of the beam in arbitrary patterns. Alternatively, the DMD could bypassed by a couple of dielectric mirrors (M1 and M2 – dielectric mirrors from Thorlabs) to maintain the gaussian profile of the beam. A dielectric mirror M3 conveys the laser beam along a path where a second beam expander (BE2) composed by lenses L3 (focal length 150 mm) and L4 (focal length 150 mm) allows to adjust the divergence of the beam before the BS that deflects part of the beam toward OBJ that focuses it on the sample (laser patterns from the projector are reported in Figure S5, Supporting Information). The gaussian beam used for the point‐like stimulation has a beam diameter of 40 µm. Between L3 and L4 a programmable shutter (SH – from Thorlabs) blocks the beam when needed.

### Modeling Visual Motion Perception

The effect of a light spot moving in on the PAZO surface was modeled by using the bi‐exponential equations described in the Note S1 (Supporting Information). The time constants have been extracted by the potentiation and relaxation profile reported in Figure S13 (Supporting Information) and in the model it was assumed that the time constants do not depend on the stimulation duration. A 500 × 500 points matrix was defined and initialized at zero intensity. The moving object was modeled by a circular mask: each point within the mask undergoes potentiation described by Equation (1) of Note S1 (Supporting Information), while each point outside the mask relaxes according to Equation (2) of Note S1 (Supporting Information). As boundary conditions, it impose for each pixel continuity in the PSI temporal responses (full Video available as Video S4, Supporting Information).

### In Materia Reservoir Computing—4‐Bit Sequences Encoding

The bit sequences are encoded in a temporal sequence of linearly polarized light pulses (P = 8.3 nW µm^−2^). Each bit has a timeframe width of 1s: when the bit is a ‘1′ a light pulse 500 ms was applied, whereas ‘0′ bit no pulse was applied. The pulses illuminate a single spot of the sample, and after each sequence, a pulse of 60 s with circular polarization erases the information and resets the material to its pristine state. All the 4‐bit sequences (16 in total) are sent 10 times on the same region of the azopolymer region to consider the cycle‐to‐cycle variability on the PAZO reservoir response. Note that in our proof‐of‐concept the device‐to‐device variability was not considered. The PSI output (reservoir output) was evaluated 100 ms after the last bit.

### In Materia Reservoir Computing—MNIST Dataset Pre‐Processing

The MNIST dataset^[^ ^75^ ^]^ for the handwritten digit recognition contains 60.000 digits for training and 10.000 digits for testing. Each digit is an 8‐bit 28 × 28 pixels grayscale image. In our experiment each image was binarized by dividing the grey scale value of each pixel by 256 and rounding the resulting number to the closest integer, so that values higher than 128 become ‘1′ and the lower ones become ‘0′. The image was then chopped in 7 matrices of 28 rows by 4 columns and merged vertically in a single matrix of 196 rows by 4 columns. In this way, each row represents a single input sequence of 4 bits that was transformed through the reservoir in a 196 × 1 array representing the reservoir output.

### In Materia Reservoir Computing—Readout

The readout was realized by a single layer fully connected neural network (196 × 10) implemented in software. For each of the 196 sequences of 4 bits representing the input digit, a PSI output value was randomly chosen among the ten possible values to form a vector of 196 components that was exploited as readout input. This neural network was offline trained through supervised learning by minimizing the categorical cross‐entropy loss function (L) implemented with Keras package in Python:

(1) $$L=-\sum_{i=1}^{N}y_{i}log\left({\hat{y}}_{i}\right)$$

where N is the dimension of the output size (in this case 10), $y_{i}$ is the target value and ${\hat{y}}_{i}$ is the read‐out model prediction. The loss function minimization was achieved through the Adam algorithm. This algorithm optimizes a stochastic gradient descent method by adaptively tuning the learning rates for the various parameters during training. These parameters are updated at each step t according to:

(2) $$w_{t+1}=w_{t}-\frac{\eta }{\sqrt{{\hat{v}}_{t}}+\varepsilon }{\hat{m}}_{t}$$

where ${\hat{m}}_{t}$ and ${\hat{v}}_{t}$ correspond to the bias‐corrected estimates of first and second moments of the gradients respectively, η and ε are parameters. This neural network was trained by using the first 60000 images as the training set and the following 10000 images as test set to evaluate the accuracy of the system.

### In Materia Reservoir Computing—Evaluation of Accuracy Variability

The effect of the variability of PAZO response on the variability of the accuracy of the MNIST task was evaluated by repeating the task 1000 times. For each iteration, the system was trained by assigning at each bit‐sequence composing the chopped and merged digit a PSI output randomly chosen among the ten experimental values. The random choice was made by changing the random seed before each iteration.

## Conflict of Interest

The authors declare no conflict of interest.

## Supporting information



Supporting Information

Supplemental Video 1

Supplemental Video 2

Supplemental Video 3

Supplemental Video 4

## Data Availability

The data that support the findings of this study are available on Zenodo (https://doi.org/10.5281/zenodo.15099683). All other data are available from the authors. The codes used to generate datasets of simulations can be accessed on GitHub (https://github.com/matero81/PAZO‐Reservoir‐Computing). The link should be now available.
