# Supplementary material for: Neuromorphic Light‐Responsive Organic Matter for in Materia Reservoir Computing
Source: Adv Mater. 2025 May 13;37(41):2501813. doi: 10.1002/adma.202501813 (PMC12531735; doi:10.1002/adma.202501813)
Supplement: Supplementary file 1 — Supporting Information [file ADMA-37-2501813-s002.docx]

Neuromorphic light-responsive organic matter for in materia reservoir computing – Supplementary Information

Federico Ferrarese Lupi, Mateo Rosero-Realpe, Antonio Ocarino, Francesca Frascella, Gianluca Milano^*^ and Angelo Angelini^*^

F. Ferrarese Lupi, M. Rosero Realpe, G. Milano, A. Angelini

Advanced Materials Metrology and Life Science Division, INRiM (Istituto Nazionale di Ricerca Metrologica), Strada delle Cacce 91, Torino 10135, Italy.

E-mail: [g.milano@inrim.it](mailto:g.milano@inrim.it), [a.angelini@inrim.it](mailto:a.angelini@inrim.it)

M. Rosero Realpe, A. Ocarino, F. Frascella

Department of Applied Science and Technology (DISAT), Politecnico di Torino, C.so Duca degli Abruzzi 24, Torino 10129, Italy.

Keywords: light-responsive polymers, neuromorphic materials, adaptive materials, in-materia reservoir computing

**
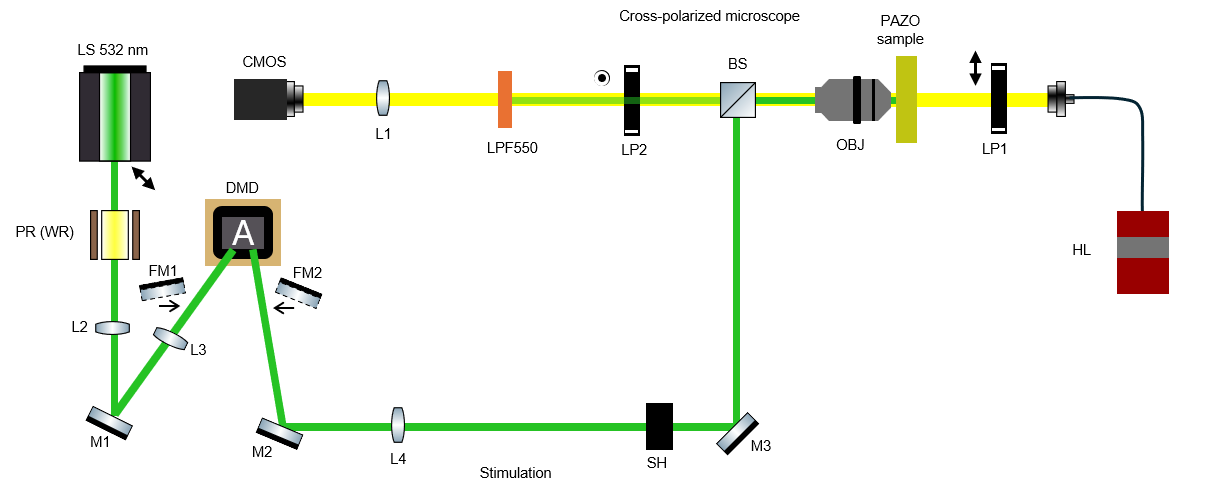
**

**Supplementary Figure S1. | Experimental setup.** A complete description of the setup is reported in the Materials and Methods section


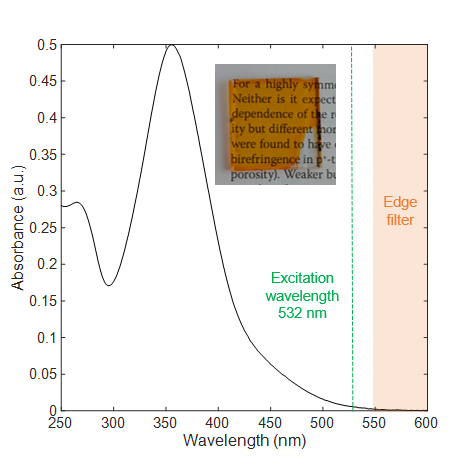


**Supplementary Figure S2. | Absorption spectrum of PAZO.** The green dashed line indicates the stimulation wavelength, the orange shadowed region indicates the spectral range of light read at the CCD camera. In the inset, a picture of PAZO spin coated on a glass substrate showing the transparency of the sample.


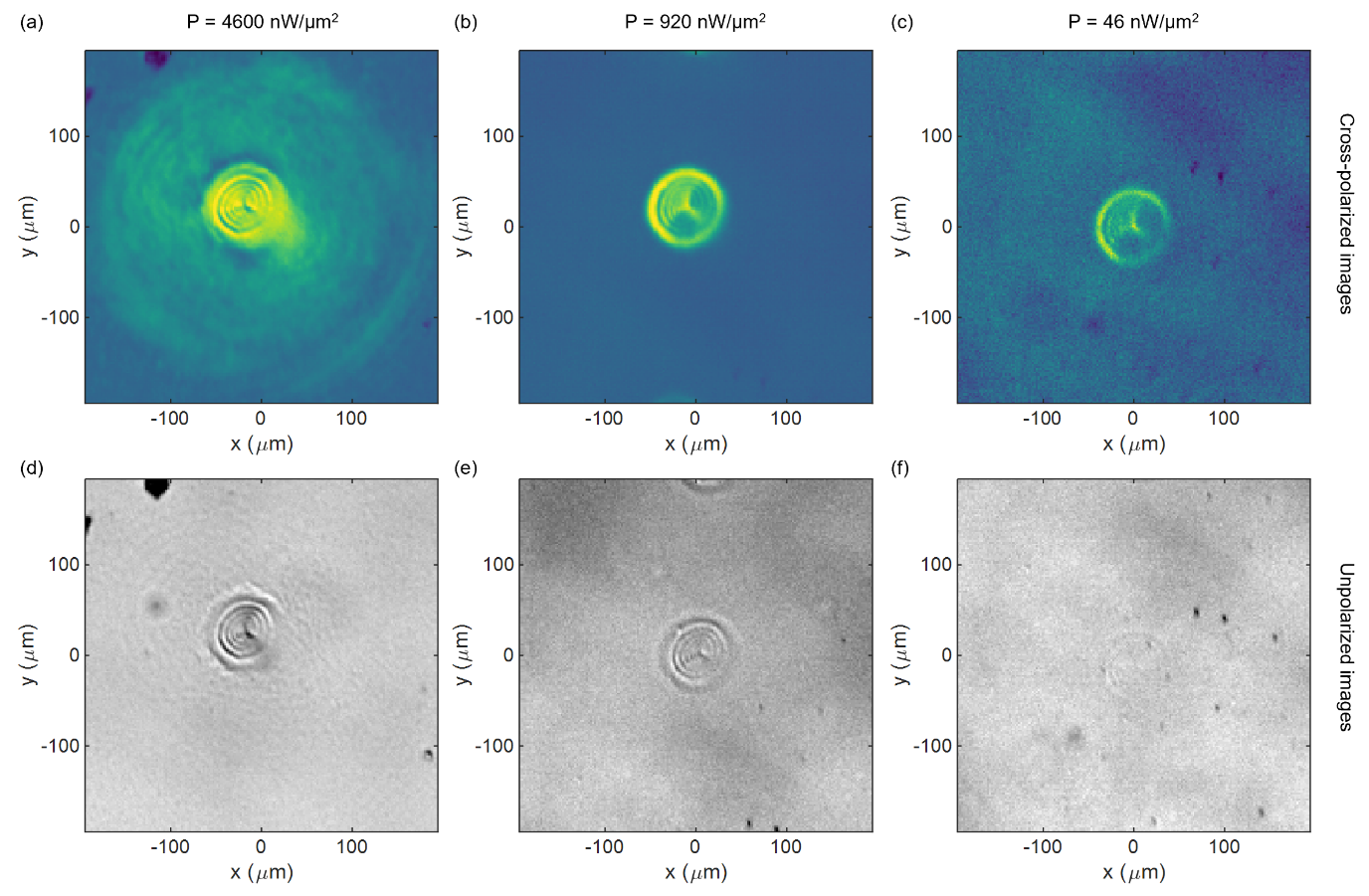


**Supplementary Figure S3 | PAZO mass migration threshold |** (a), (b) and (c) are cross-polarized images showing the birefringence in the illuminated area. (d), (e) and (f) are the corresponding white light images where the surface modification due to light-driven mass migration can be observed. In our experiments we used power densities (8.3 nW/μm^2^ for single-point experiments) that do not induce significant mass-migration.


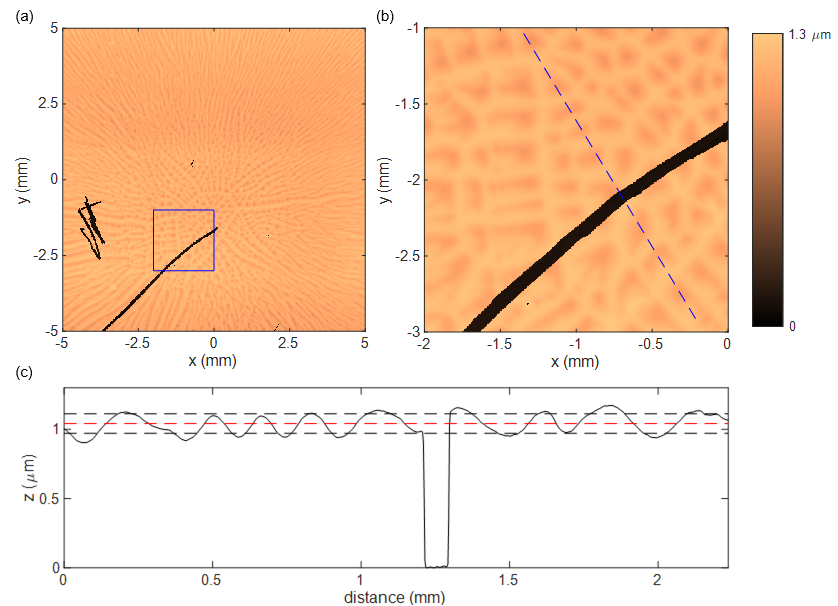


**Supplementary Figure S4. | PAZO thin film topographic characterization |** (a) False color map of the surface topography measured by an optical profilometer over a 1 x 1 cm area. (b) Detail of the surface extracted from the blue square in (a) containing an engraving in the PAZO film. (c) Depth profile of the thin film extracted along the blue dashed line in (b). The red dashed line is the mean film thickness, the black dashed lines indicate the standard deviation.


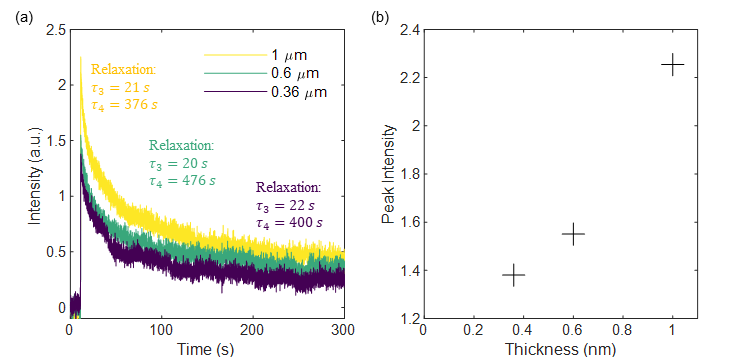


**Supplementary Figure S5 | PAZO response for different film thicknesses |** (a) Time-evolution of the transmitted light intensity for different PAZO film thicknesses when stimulating the material with a single pulse (λ = 532nm, P = 30 nW/µm^2^, t_ON_ = 200 ms). (b) Peak intensity as a function of the film thickness.


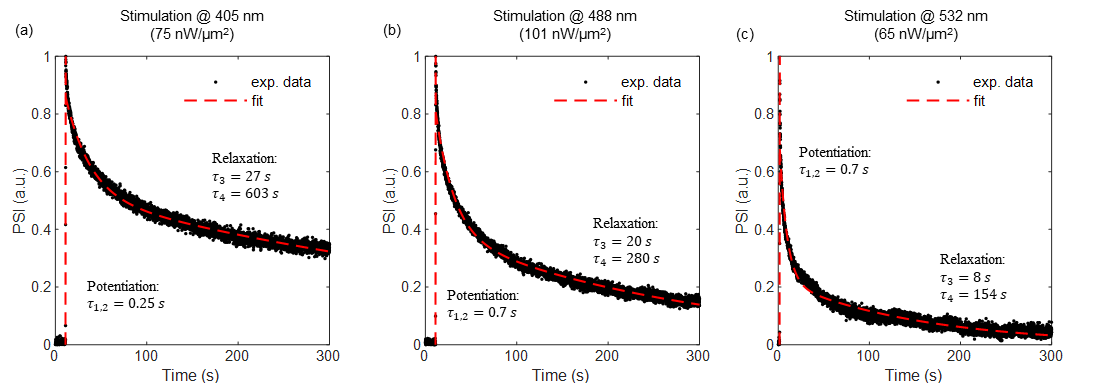


**Supplementary Figure S6. | Birefringence dynamics at different excitation wavelengths.** Single pulse stimulation (500 ms) at (a) 405 nm (75 nW/µm^2^), (b) 488 nm (101 nW/µm^2^) and (c) 532 nm (65 nW/ µm^2^). The black dots indicate the experimental data, while the red dashed line is a double exponential step function for potentiation and relaxation (see equations (1) and (2) in Supplementary Note N1.


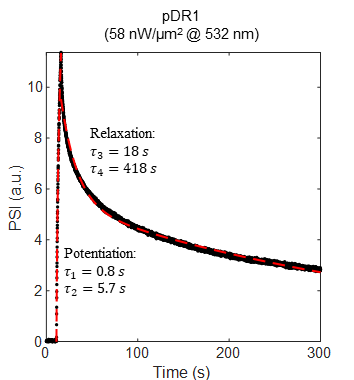


**Supplementary Figure S7. pDR1M optical response |** Time-evolution of the transmitted light intensity for pDR1M when stimulating the material with a single pulse (λ = 532nm, P = 58 nW/µm^2^, t_ON_ = 5 s). The table on the right reports the decay constants for potentiation and relaxation.


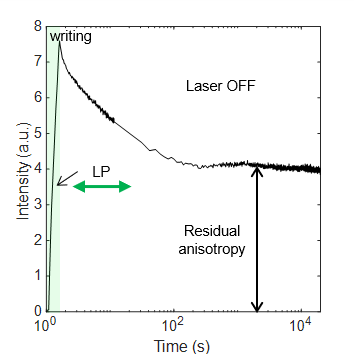


**Supplementary Figure S8. | Retention.** Linearly polarized stimulation (1 second pulse, 8.3 µW/µm^2^) and spontaneous relaxation. The x axis is in logarithmic scale for better visualization.


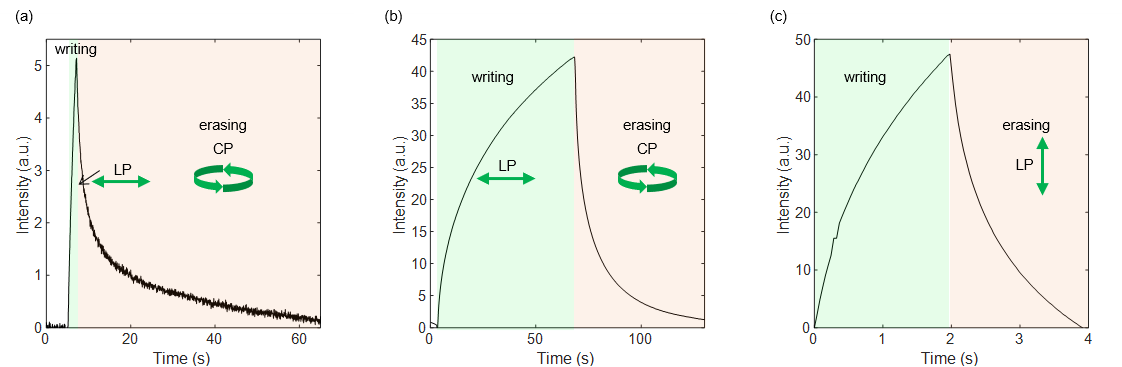


**Supplementary Figure S9. | Stimulation and erasing processes.** (a) Linearly polarized stimulation (1 second pulse, 8 nW/µm^2^) and erasing with circular polarization. (b) Linearly polarized stimulation (60 seconds pulse, 8 nW/µm^2^) and erasing with circular polarization. (c) Linearly polarized stimulation (2 seconds pulse, 30 nW/µm^2^) and erasing with orthogonal polarization.


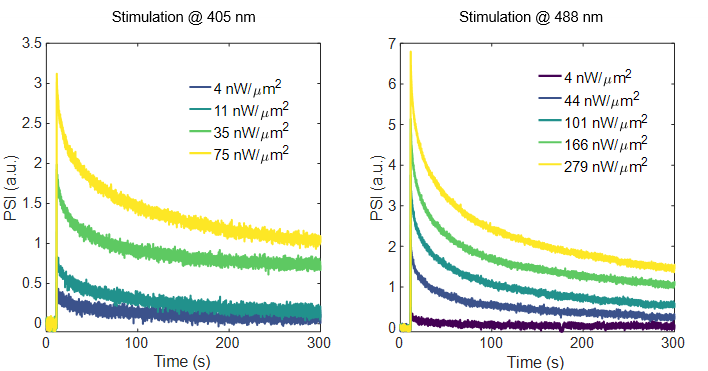


**Supplementary Figure S10. | Potentiation at different wavelengths.** Single pulse (500 ms) potentiation and relaxation dynamic when PAZO is stimulated at (a) 405 nm and (b) 488 nm at different power densities.


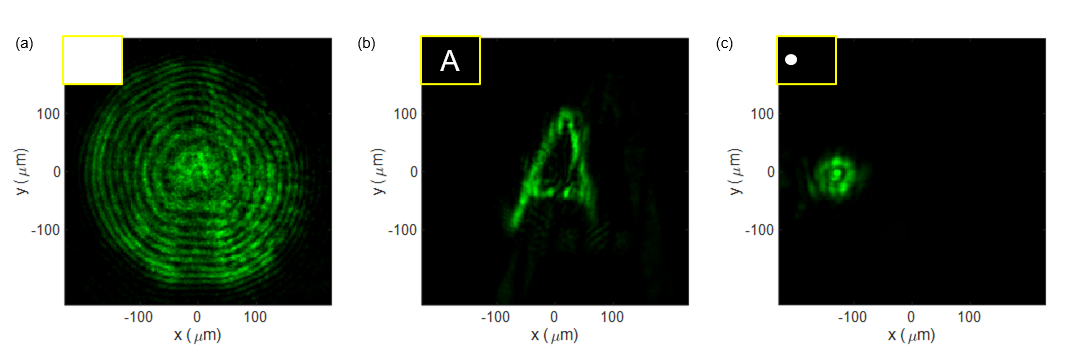


**Supplementary Figure S11. |** **Stimulation patterns.** The laser patterns emerging from the DMD and projected on the PAZO sample. The corresponding input patterns are reported in the insets: (a) The full screen reflects the gaussian beam. (b) Projection of the letter ‘A’ in the center of the screen. (c) A single point placed in a chosen position.


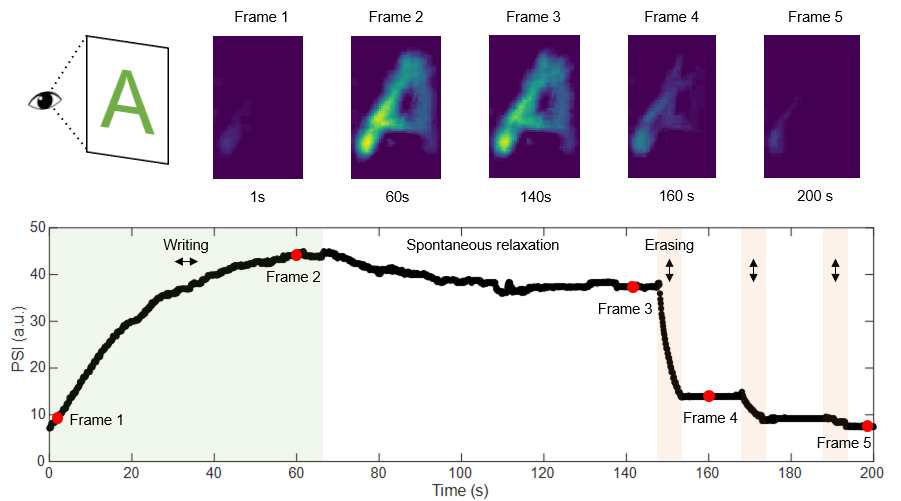


**Supplementary Figure S12. |** **Writing and erasing dynamics of a spatially distributed light intensity pattern.** A light intensity pattern corresponding to the letter ‘A’ is used as a pre-synaptic stimulation. The continuous exposition in the first 60 seconds enhances the PSI of the whole pattern (Frame 1-2). After the stimulus is turned OFF, spontaneous relaxation weakens the PSI to a stationary level corresponding to a LTM status (Frame 4). By applying stimulation with orthogonal polarization the PSI can be lowered to an intermediate value (Frame 4) where the pattern is still visible, or to erase it completely (Frame 5). Supplementary Video V2 shows the full process.


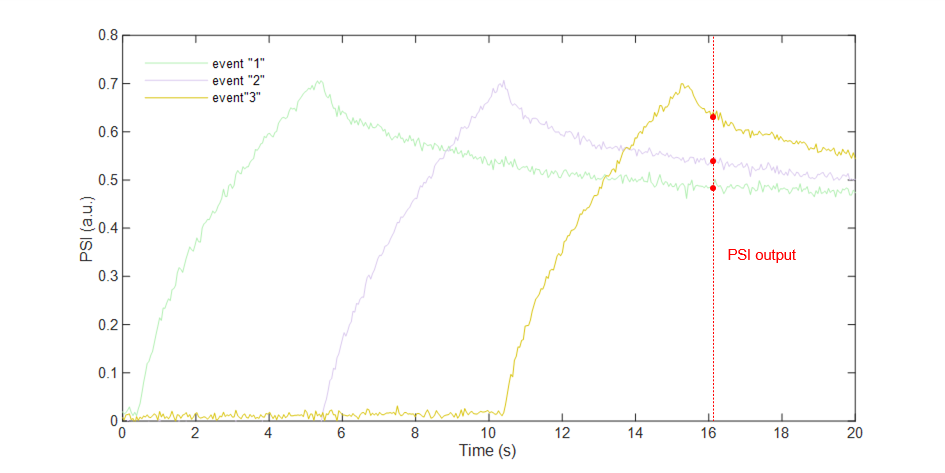


**Supplementary Figure S13. |** **Temporal trace of spatio-temporal uncorrelated events.** PSI temporal profile of three events recorded in distinct positions. Each event corresponds to a local stimulation (t_ON_ = 5 s) of a gaussian beam (P = 0.15 µW/µm^2^, spatial profile in Fig. S5c). The PSI read 1 second after the last event allows to retrieve the temporal sequence of the uncorrelated events.


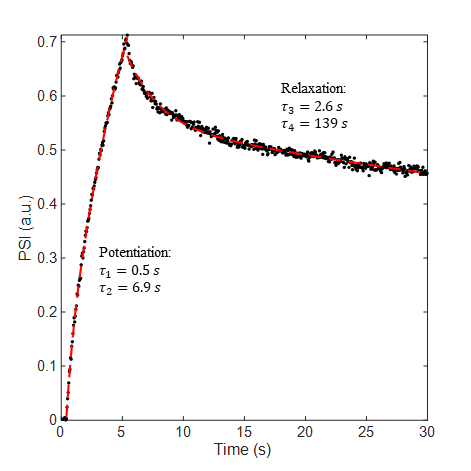


**Supplementary Figure S14. PAZO dynamics for long time exposure |** Potentiation under continuous irradiation for 5 s and relaxation dynamics. The experimental data are the same reported in Supplementary Figure S12 and labeled as Event 1. The table on the right reports the decay constants resulting from the fit.

**
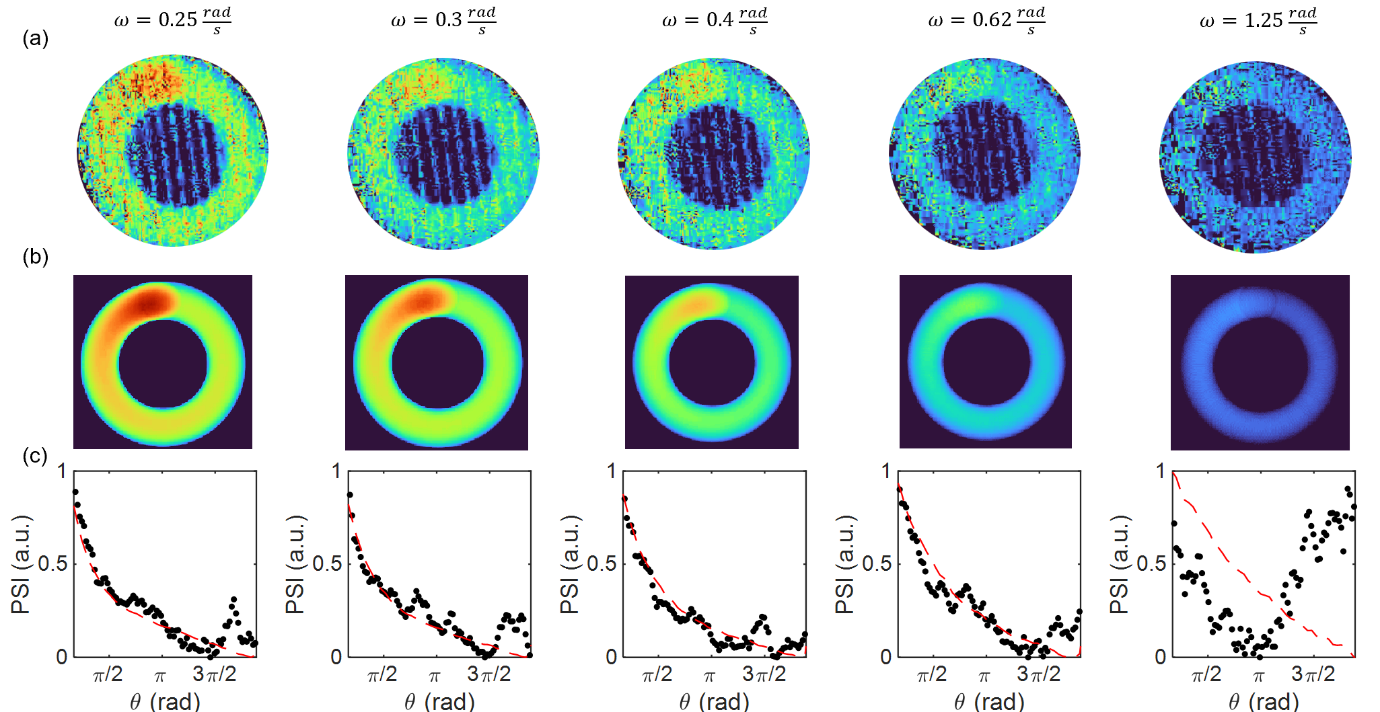
**

**Supplementary Figure S15. | Experimental and modeled motion perception |** (a) From left to right, false color PSI images of clockwise continuously moving stimulation at different angular speeds. (b) Corresponding modeled PSI images. To model potentiation and relaxation at each pixel, we used the time constants extracted from the experimental data in Supplementary Figure 7 by using the bi-exponential equations reported in Supplementary Note N1.(more details in the Methods section. (c) PSI profiles extracted along the experimental (black dots) and modeled (red dashed line) trajectories.

**
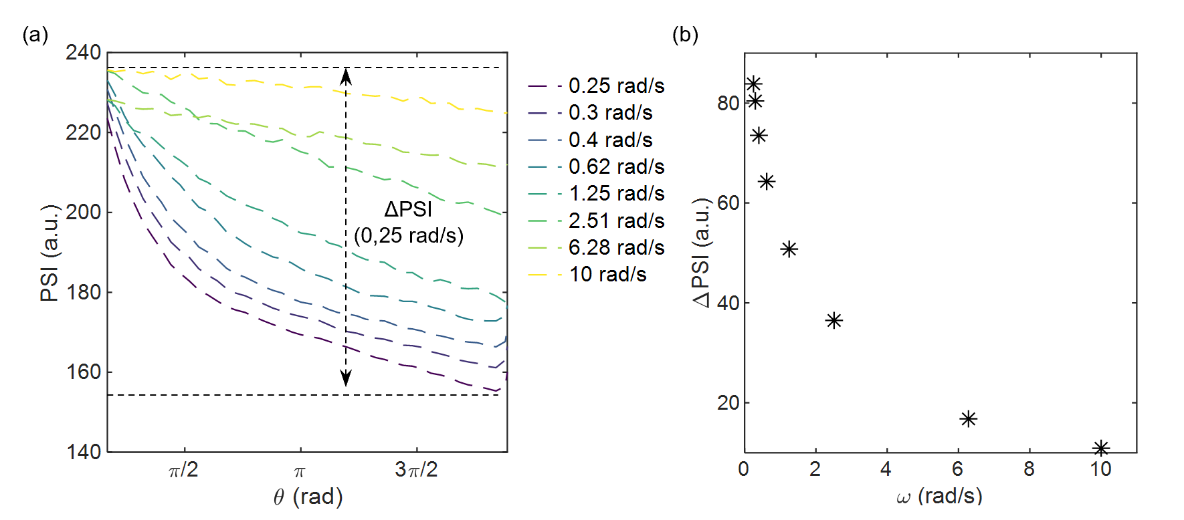
**

**Supplementary Figure S16. | Simulated PSI signal of moving objects at different speeds |** (a) PSI extracted along the trajectory of an object moving at different speeds. (a) Difference of PSI intensity between the first and the last point of the trajectory for different angular speeds.


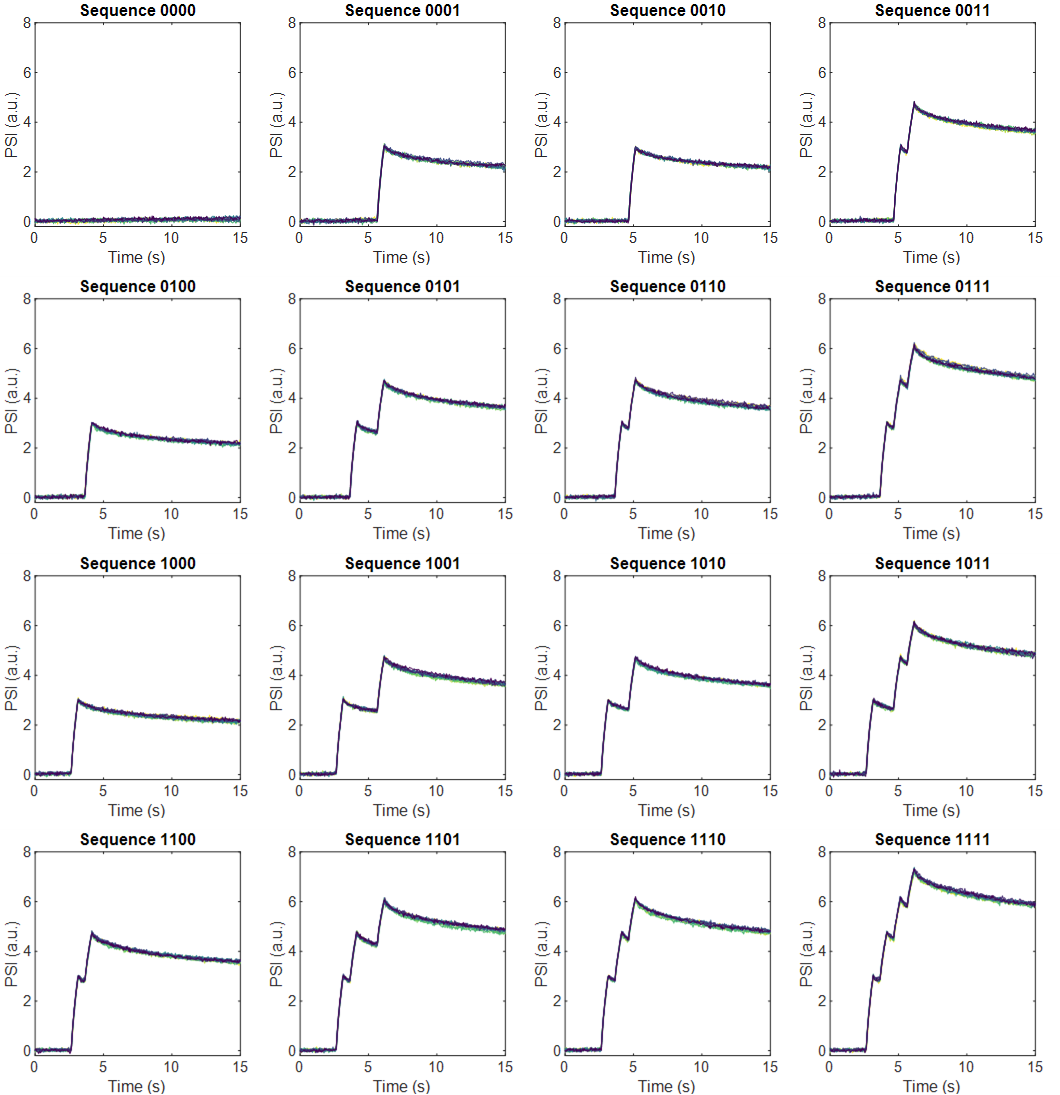


**Supplementary Figure S17. |** **Cycle-to-cycle variability.** PSI temporal profile under different stimulation pulse sequences. Each sequence is repeated 10 times. After each sequence, the internal state is of PAZO is resetted to its pristine state by actively erasing the information with a circularly polarized laser beam.


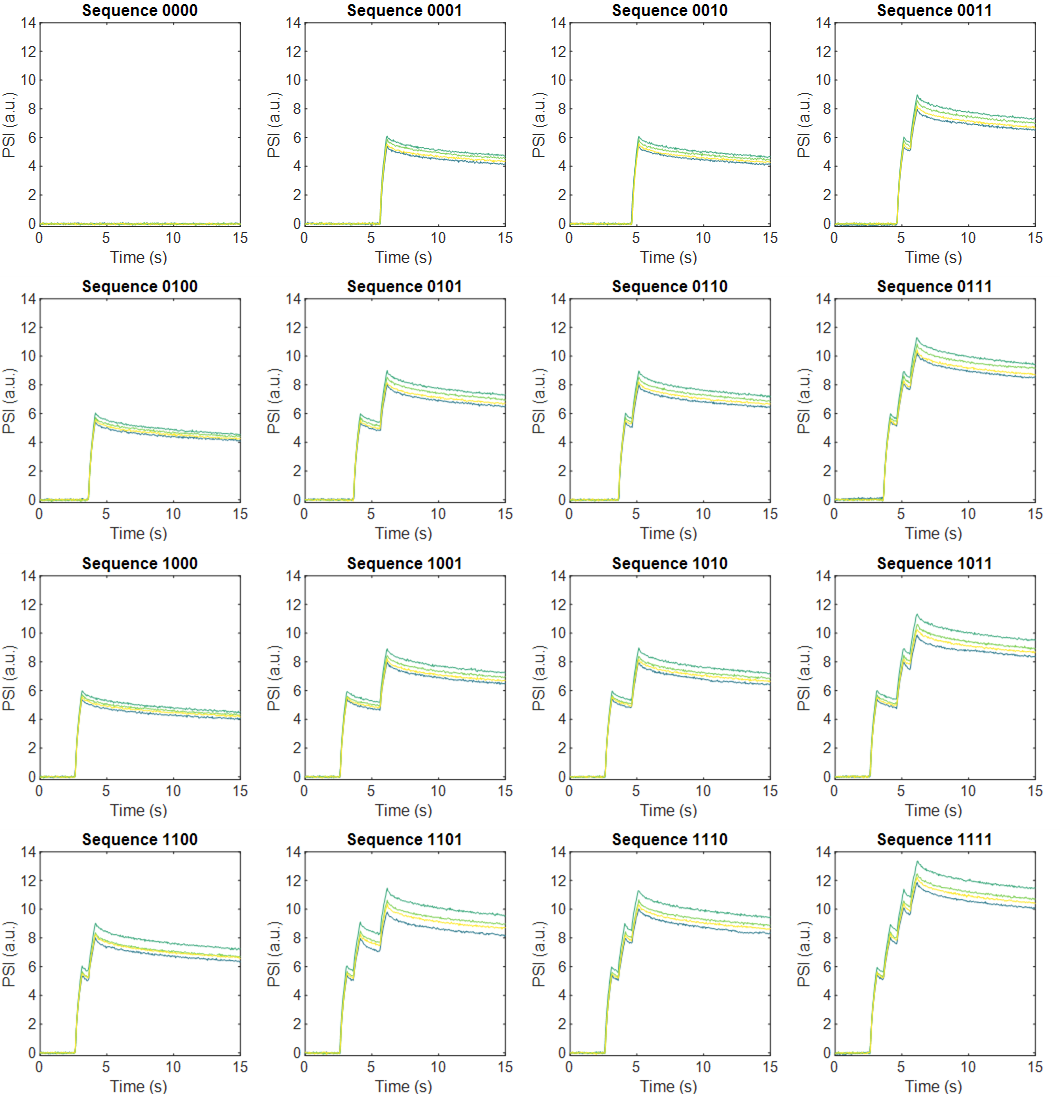


**Supplementary Figure S18 | Device-device variability.** Each sequence is repeated in four different positions on the sample. After each sequence, the internal state is of PAZO is resetted to its pristine state by actively erasing the information with a circularly polarized laser beam.


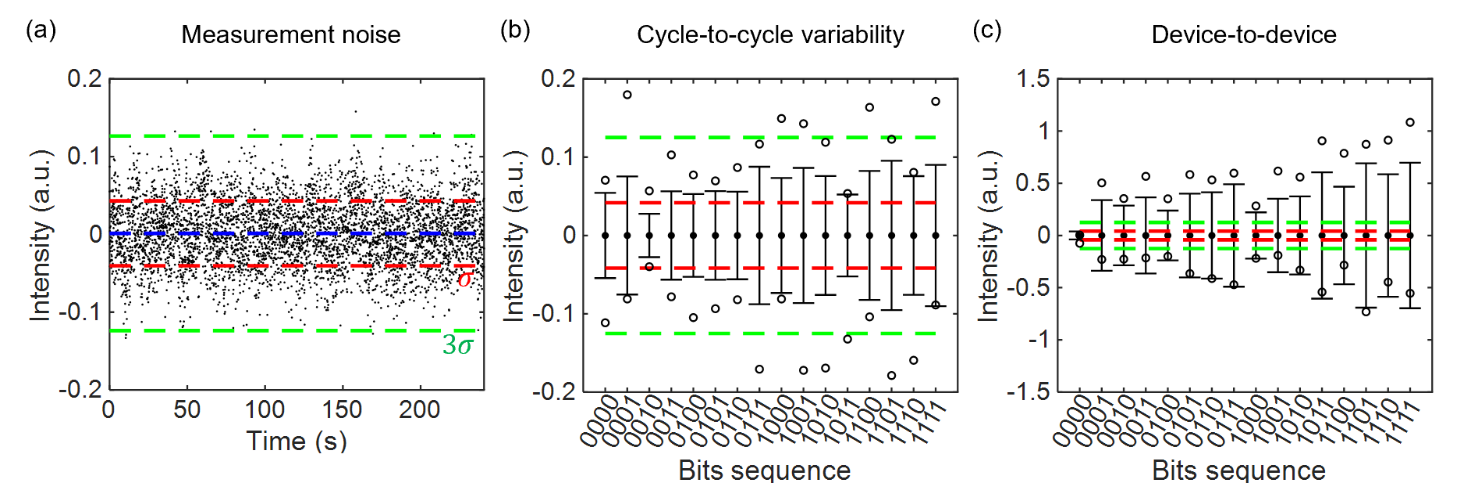


**Supplementary Figure S19 | Experimental data dispersion.** (a) Measurement noise**.** The red dashed lines and the green dashed lines indicate the standard deviation 𝜎 and the 3𝜎 values around the mean value (blue dashed line). (b,c) Cycle-to-cycle (b) and device-to-device (c) experimental data dispersion. The black whiskers and the black circles indicate the 𝜎 and the maxima/minima of the PSI output associated to the experimental data in Supplementary Figure S17 and Supplementary Figure S18 respectively.


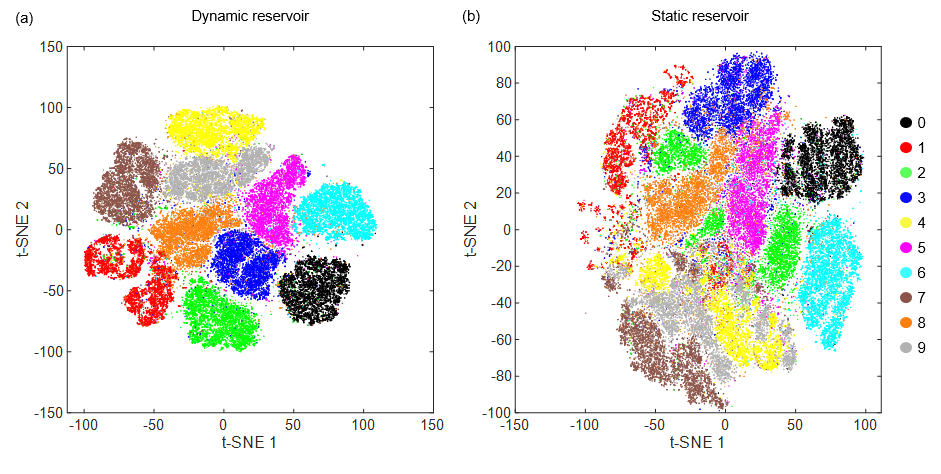
**Supplementary Figure S20. |** **Comparison of data clusterization whit t-SNE algorithm.** (a) Data clusterization when PAZO is used as dynamic reservoir and the 196 reservoir states are used as input of the t-SNE algorithm. (b) Data clusterization when a static reservoir is considered. The static reservoir is obtained by using the last bit of each one of the 196 rows as input to the t-SNE algorithm so that the data dimension in the two cases is the same.


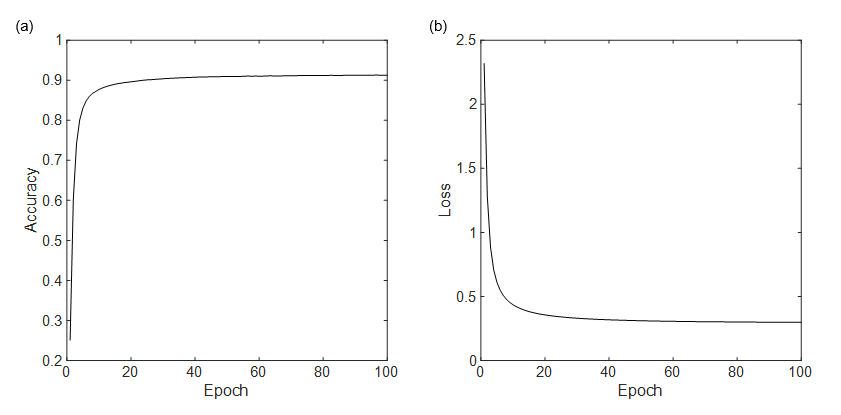


**Supplementary Figure S21. |** **Training functions of the MNIST dataset** (a) Accuracy function versus epochs. (b) Loss function versus epochs.


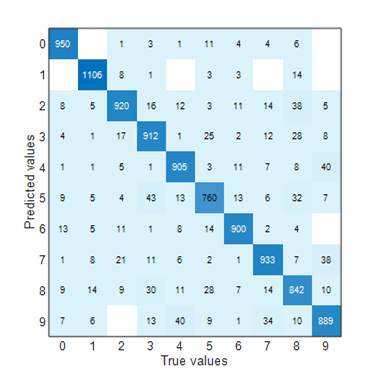


**Supplementary Figure S22. |** **Confusion Matrix of the predicted values of 10.000 handwritten digits from the MNIST dataset.** The confusion matrix reports the occurrences of the predicted values.

**Table S1.** Accuracy comparison of MNIST recognition task using physical systems as reservoir and a single layer ANN as readout.

| Reference | Device | Image Size | Pre-processing | Readout | Accuracy |
| --- | --- | --- | --- | --- | --- |
| This work | Optical  PAZO | 28x28 | Binarize  Chop & Merge (196x4) | 196x10 | 91.4% |
| Software  (Single layer ANN) | - | 28x28 | Binarize | 784x10 | 91.7% |
| Du et al.  *Nat. Comm.* (2017)^1^ | Memristor  Pd/WO_x_/W | 22x20 | Binarize  Chop & Merge (88x5)^a^ | 176x10 | 88%^b^ |
| Midya et al.  *Adv. Intell. Syst.* (2019)^2^ | Memristor Pd/Ag/SiO_2_/Pt | 22x20 | Binarize  Chop & Merge (110x4) | 110x10 | 83% |
| Milano et al.  *Nat. Mater.* (2022)^3^ | Memristor  Ag/PVP/Ag | 28x28 | Binarize  Chop & Merge (196x4) | 196x10 | 90.4% |
| Woo et al.  *ACS Nano* (2024)^4^ | Memristor  Cu_0.1_Te_0.9_/HfO_2−x1_/  HfO_2−x2_/Pt | 28x28 | Binarize  Chop & Merge (196x4) | 196x10 | 91.3% |
| Ghenzi et al.  *Nanosc. Horiz.* (2023)^5^ | Memristor  Ta_2_O_5_/HfO_2_ | 28x28 | Binarize  Chop & Merge (196x4) | 196x10 | 91% |
| Jang et al.  *Nat. Comm.* (2021)^6^ | Memristor  W/HfO_2_/TiN | 28x28 | Binarize  Chop & Merge (196x4) | 196x10 | 90% |
| Zha et al.  *Adv. Mater.* (2023)^7^ | Optoelectronic  Te/CuInP2S6  VdW Heterostructure | 22x20 | Binarize  Chop & Merge (110x4) | 110x10 | 80% |

^a^ In this case the input is composed by 5-bit sequences. In all the other works in the table the input is composed by 4-bit sequences.

^b^ In this case each bit sequence composing the image was encoded in input pulse trains with two different pulse rates


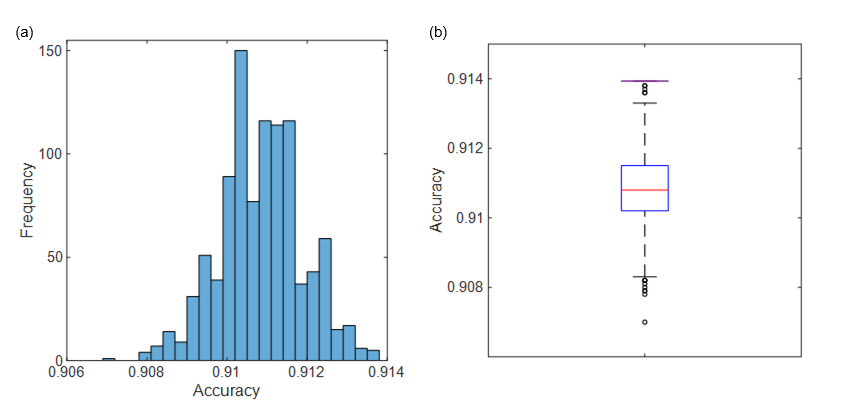


**Supplementary Figure S23. |** **Accuracy variability.** (a) Accuracy distribution of the MNIST dataset over 1000 trainings with random choice of the experimental data according to the algorithm in Fig. S8 and (b) the corresponding boxplot where the red bar is the mean accuracy, the blue box comprises the upper and lower quartile, the whiskers extend to the minimum and maximum accuracy values that are not outliers and the black circles are the outliers. The purple bar above is the accuracy value obtained using the mean value of the 10 experimental PSI values. Note that the accuracy of the 196 inputs not processed by the dynamic reservoir (static reservoir) is 84.8%.

**Supplementary Note N1 | Light-induced birefringence dynamics.**

To analyze the potentiation and relaxation dynamics we stimulated the material with a light pulse at three distinct wavelengths (405 nm, 488 nm and 532 nm, Supplementary Figure S4) and fitted the light intensity temporal profile I(t) with a double exponential function for potentiation (1) and relaxation/erasing (2):

$I\left( t \right)= I_{sat}(1-Ae^{-\frac{t}{\tau_{1}}}-Be^{-\frac{t}{\tau_{2}}})$ (1)

$I\left( t \right)=Ce^{-\frac{t}{\tau_{3}}}+De^{-\frac{t}{\tau_{4}}}$ (2)

where $I_{sat}$ is the saturation intensity, A, B, C and D are the weights of the exponential terms and $\tau_{i}$ are the time constants. The results of the fitting procedure, including the coefficient of determination R^2^ are reported in Supplementary Table T1.

The results show that for short pulse excitation, as in our case, the potentiation process is well described a single exponential function. The relaxation process contains the two contributions, and for all the three wavelengths we observe roughly an order of magnitude of difference between the time constants, as also observed by Song and co-workers.^8^

By comparing the results between the different wavelengths, we observe that excitation with longer wavelengths results in smaller time constants (Supplementary Figure S6).

**References**

1. Du, C. *et al.* Reservoir computing using dynamic memristors for temporal information processing. *Nat Commun* **8**, 2204 (2017).
2. Midya, R. *et al.* Reservoir Computing Using Diffusive Memristors. *Advanced Intelligent Systems* **1**, 1900084 (2019).
3. Milano, G. *et al.* In materia reservoir computing with a fully memristive architecture based on self-organizing nanowire networks. *Nat Mater* **21**, 195–202 (2022).
4. Woo, K. S. *et al.* Memristors with Tunable Volatility for Reconfigurable Neuromorphic Computing. *ACS Nano* **18**, 17007–17017 (2024).
5. Ghenzi, N. *et al.* Heterogeneous reservoir computing in second-order Ta2O5/HfO2 memristors. *Nanoscale Horiz* **9**, 427–437 (2023).
6. Jang, Y. H. *et al.* Time-varying data processing with nonvolatile memristor-based temporal kernel. *Nat Commun* **12**, 5727 (2021).
7. Zha, J. *et al.* Electronic/Optoelectronic Memory Device Enabled by Tellurium-based 2D van der Waals Heterostructure for in-Sensor Reservoir Computing at the Optical Communication Band. *Advanced Materials* **35**, 2211598 (2023).
8. Song O.–K. *et al.* Dynamic Processes of Optically Induced Birefringence of Azo Compounds in Amorphous Polymers below *T*g. *Macromolecules* **30**, 6913–6919 (1997).
